# The Impact of Hospital-Based Fidaxomicin Use Criteria on *Clostridioides difficile* Recurrence: A Retrospective Matched Cohort Study

**DOI:** 10.3390/idr18040090

**Published:** 2026-08-19

**Authors:** Song Kwon, Rachel Gabor, Philip Whitfield

**Affiliations:** 1 Department of Pharmacy, Marshfield Clinic Region–Sanford Health, Marshfield, WI 54449, USA; skwon2675@gmail.com; 2 Biostatistics, Marshfield Clinic Health System and Marshfield Clinic Research Institute, 1000 North Oak Avenue, Marshfield, WI 54449, USA; rachel.gabor@sanfordhealth.org

**Keywords:** *C. diff*, *Clostridioides difficile*, fidaxomicin, vancomycin, restriction, antimicrobial, expenditure

## Abstract

Background/Objectives: *Clostridioides difficile* infection (CDI) is associated with substantial morbidity and high recurrence rates. Current guidelines recommend fidaxomicin over oral vancomycin due to reduced recurrence; however, its high acquisition cost limits widespread adoption. Our health system implemented criteria restricting fidaxomicin to patients with the highest risk for recurrence. This study evaluated the impact of this strategy on CDI recurrence and antimicrobial expenditures. Methods: We conducted a multicenter, retrospective matched cohort study across a rural health system from January 2023 through March 2026. The fidaxomicin criteria for use (intervention) went into effect on 1 January 2025. Adult hospitalized patients with an initial CDI episode treated with oral vancomycin and/or fidaxomicin were included. Patients with severe CDI or prior CDI episodes were excluded. A 2:1 matching algorithm based on key recurrence risk factors was used to compare pre-implementation (before criteria for use) and post-implementation (after criteria for use) cohorts. The primary outcomes were 28-day and 90-day CDI recurrences. Secondary outcomes included antimicrobial utilization and annualized expenditures. Results: A total of 144 matched patients were analyzed (96 control and 48 intervention). Fidaxomicin use decreased from 40.6% to 8.3% following implementation (*p* < 0.001). Annualized fidaxomicin expenditures declined by 54.7%. CDI recurrence rates were similar between groups: 28-day recurrence was 5.2% in the control group versus 6.2% in the intervention group, and 90-day recurrence was 7.3% versus 10.4%, respectively, with statistical non-inferiority at 28 days (*p* = 0.014). Conclusions: Implementation of risk-based fidaxomicin use criteria significantly reduced utilization and costs without a statistically significant increase in CDI recurrence. These findings support a targeted stewardship approach to optimize resource use while maintaining comparable clinical outcomes.

## 1. Introduction

*Clostridioides difficile* infection (CDI) poses a substantial public health concern in both hospital and community settings, contributing significantly to patient morbidity and healthcare utilization [1]. Recurrence rates are of particular concern for CDI management: following an initial episode, approximately 20–30% of patients experience a first recurrence, and the risk of recurrence increases substantially with each subsequent episode, reaching 40–60% in patients with second and subsequent recurrences [2,3,4]. Other host risk factors for an initial recurrence include advanced age, immunocompromised status, antibiotic exposure, and gastric acid suppression [5,6,7,8,9]. Recurrent CDI (rCDI) places patients at heightened risk for prolonged illness, repeated hospitalizations, decreased quality of life, and mortality [2].

Fidaxomicin has minimal activity against much of the normal gut microbiota, preserving microbial diversity and colonization resistance, and may also inhibit *C. difficile* sporulation [10,11]. These properties likely contribute to the reduced risk of recurrent CDI observed with fidaxomicin compared with oral vancomycin, despite comparable initial cure rates [10]. Consequently, the 2021 SHEA/IDSA guidelines suggest fidaxomicin over oral vancomycin for CDI treatment [12].

However, the high acquisition cost of fidaxomicin places a significant financial burden on health systems [13]. Patient procurement at discharge similarly presents a barrier—the same IDSA/SHEA guidelines acknowledge that the average wholesale price has remained unchanged since the drug’s launch in 2011. As healthcare systems face mounting pressure to simultaneously maintain affordability and quality of care, unique strategies are needed to control costs while providing patients with optimal, guideline-concordant care.

At our large rural Wisconsin health system, fidaxomicin represented the highest antibiotic expenditure several years running and additionally coincided with multiple incidents of patients being unable to afford fidaxomicin following discharge. In response, the fidaxomicin criteria for use were created, with the hypothesis that not all patients are at the same risk of rCDI, and with the criteria based on the available literature, fidaxomicin use and expenditure may be reduced without increased rates of rCDI. Given the potential risk for fidaxomicin underutilization, the clinical impacts of these prescribing changes need to be evaluated. This study aims to compare CDI recurrence rates at 28 and 90 days before and after implementation of the new fidaxomicin criteria for use. The results may also inform optimal fidaxomicin utilization strategies, ensuring that patients at the highest risk derive maximal clinical benefit while maintaining responsible stewardship of healthcare resources.

## 2. Materials and Methods

This multicenter, retrospective observational cohort study was conducted across the Marshfield Region of Sanford Health (Marshfield, WI, USA), a rural health system comprising 11 hospitals. The study protocol was reviewed by the local institutional review board and deemed exempt. Data was retrieved from the electronic health record (EHR) from 1 January 2023 to 31 March 2026. Eligibility criteria included patients 18 years of age or older with their first positive *Clostridioides difficile* nucleic acid amplification test (CDNAAT) 48 h before or after hospitalization that were treated with either vancomycin 125 mg by mouth four times daily or 200 mg of fidaxomicin by mouth twice daily. Patients were assigned to one of two cohorts based on the timing relative to the implementation of the fidaxomicin criteria for use. The control cohort consisted of patients treated before 1 January 2025, and the intervention cohort consisted of patients treated on or after 1 January 2025, that is, after the criteria became active.

Key exclusion criteria included diagnosis of severe CDI identified through ICD-10 codes indicating toxic megacolon or ileus (K59.31 or K56), treatment with IV metronidazole, rectal vancomycin, or oral vancomycin doses greater than 125 mg four times daily, and ICD-10 code for rCDI (A04.71) at any time prior to the index hospitalization. Patients with no follow-up visits within our health system or mortality within 90 days of the index hospitalization were considered lost to follow-up. Outcomes of this study included 28-day and 90-day rCDI. These were identified through another positive CDNAAT and antimicrobial treatment for CDI in the 28 or 90 days following the end of treatment for the index case. Treatment completion was defined as the last day of CDI therapy, which was determined by combining the inpatient treatment duration with the duration of any discharge prescription. If no discharge prescription was provided, treatment completion was defined as the final day of inpatient therapy. For our study to obtain 80% power with a non-inferiority margin of 10% based on an expected baseline recurrence rate of 5%, we targeted a sample of at least 45 intervention patients and 90 control patients. A 10% non-inferiority margin was chosen as a preponderance of those involved in the development of the criteria agreed that it would be clinically meaningful, and other relevant publications have used the same margin [10]. Secondary outcomes included antimicrobial expenditure (annualized) and fidaxomicin and oral vancomycin use rates.

The fidaxomicin criteria for use were developed in collaboration with the Pharmacy Division and the Antimicrobial Stewardship Committee, with subsequent approval by the Pharmacy and Therapeutics Committee. The criteria were designed to be pragmatic and data-driven based on the best available evidence. Following approval, providers and pharmacists were educated via departmental emails and in-person departmental presentations. Next, fidaxomicin was made orderable solely through an order set including the criteria, and an alert prompting pharmacist to review patient eligibility prior to fidaxomicin verification was implemented. If fidaxomicin was ordered for a patient that did not meet the criteria, the pharmacist was to notify the ordering provider who would then either cancel the fidaxomicin and order oral vancomycin or would order a consult with an infectious disease specialist who would then determine the appropriateness of fidaxomicin use. This criterion was implemented on 1 January 2025 and may be found in its entirety in Table 1 and the entire stewardship process is shown in Supplementary Materials.

To ensure similar distributions of key drivers of CDI recurrence before and after the intervention, a 2:1 matching algorithm was employed using risk factors selected a priori for rCDI: age, immunocompromised status, and antibiotic administration during index admission. Multiple matching methodologies were evaluated (nearest neighbor matching on propensity scores, cardinality matching, optimal pair matching, and exact matching). Cardinality matching was selected to minimize standardized mean differences in matching criteria and among additional variables (sex, 30-day history of antibiotic use, and Charlson Comorbidity Score).

Patient and clinical characteristics were described using means and standard deviations and counts and percentages were used for the unmatched and matched samples. Differences in patient characteristics between the control and intervention populations were compared using *t*-tests and chi-squared tests, as appropriate. Antibiotic use rates during the control and intervention periods were reported. The primary outcome of CDI recurrence was reported at 28 days and 90 days. A non-inferiority test for proportions was used to assess whether the recurrence rate during the intervention period was non-inferior to the control period with a 10% margin of difference (using the non-inferiority (“less”) option for a difference in two proportions). All data were analyzed in R version 4.5.1 (R Core Team, Vienna, Austria) with support from open sourced TOSTER package (version 0.8.6), the MatchIt package (version 4.7.2), and the cobalt pacakge (version 4.6.2) [14,15,16,17,18].

## 3. Results

### 3.1. Patients

A total of 174 patients were included in the overall cohort, with 144 patients retained in the matched sample following 2:1 matching. Baseline characteristics were similar between the overall and matched cohorts (Table 2 and Table 3). The mean age was 67.1 ± 14.3 years in the overall cohort compared to 66.5 ± 14.8 years in the matched cohort. The sex distribution remained identical, with 50% female representation in both groups. Comorbidity burden was also comparable, with a median Charlson Comorbidity Index (CCI) of 3.0 in both cohorts. Similarly, the proportion of immunocompromised patients was unchanged (7.5% vs. 7.6%). Facility distribution remained consistent after matching, with the largest proportion of patients treated at the flagship hospital. Rates of antibiotic exposure within 30 days prior to admission were also similar between groups (44.3% vs. 39.6%). While antibiotic use during the index admission was lower in the matched cohort (33.3% vs. 23.6%), the overall characteristics remained well balanced.

Within the matched cohort, baseline characteristics were well balanced between the control (*n* = 96) and intervention (*n* = 48) groups. The mean age was similar between groups (66.3 ± 15.4 vs. 66.9 ± 13.6; *p* = 0.801). There were no statistically significant differences in sex distribution (*p* = 0.216) or facility of care (*p* = 0.322). Comorbidity burden was slightly higher in the control group (CCI 4.0 ± 3.2 vs. 3.0 ± 1.9), though this did not reach statistical significance (*p* = 0.051). The proportion of immunocompromised patients was similar between groups (7.3% vs. 8.3%; *p* > 0.99). A full account of the matched sample patient characteristics is found in Table 3.

### 3.2. Outcomes

Among the matched cohort, implementation of the fidaxomicin criteria for use resulted in a statistically significant shift in treatment patterns for the index infection. Fidaxomicin use decreased substantially from 40.6% in the control group to 8.3% in the intervention group, while oral vancomycin use increased from 59.4% to 91.7% (*p* < 0.001). This change in prescribing patterns was associated with a marked reduction in antimicrobial expenditures at the health system level. Annualized fidaxomicin expenditures decreased by 54.7%, from $165,964 in 2023 to $75,124 in 2025, while vancomycin expenditures increased by 15.8%, from $6768 to $7838. Overall, total antimicrobial spending decreased by 52.0%, representing an absolute reduction of $89,770 annually (Table 4). Notably expenditure data reflects aggregate pharmacy spending across all acute care facilities and was not limited to the study population.

Clinical outcomes were similar between groups. The 28-day recurrence rate in the control group was 5.2% compared to 6.2% in the intervention group, and 90-day recurrence occurred in 7.3% of patients in the control group and 10.4% of patients in the intervention group (Table 5). Non-inferiority testing demonstrated that the difference in 28-day recurrence rates with an absolute difference of 1% (95% CI: −6.9–9.0%, *p* = 0.014) met the predefined non-inferiority margin of less than 10%. The 90-day recurrence rate was 3.1% higher in the intervention group; however, this was not statistically significant (90% CI: −6.4–12.6%, *p* = 0.079).

## 4. Discussion

Implementation of the 2021 IDSA/SHEA guideline recommendation favoring fidaxomicin over oral vancomycin for initial CDI episodes has been inconsistent across the United States because of cost and formulary constraints [19]. In our health system, implementation of the risk-stratified fidaxomicin use criteria was associated with substantially reduced fidaxomicin utilization and lower antimicrobial expenditures with statistical non-inferiority in 28-day CDI recurrence. The absolute differences in recurrence rates between periods were small (1.0% at 28 days and 3.1% at 90 days), although the low number of recurrence events and the relatively small post-implementation cohort limit definitive conclusions regarding clinical equivalence, particularly for the 90-day recurrence rates. Collectively, these findings suggest that a risk-based stewardship strategy may reduce fidaxomicin utilization and associated costs while maintaining similar observed recurrence rates. Rather than uniformly restricting fidaxomicin use, the criteria prioritized patients with established risk factors for recurrence, including prior CDI recurrence, being severe immunocompromised, or advanced age with concomitant systemic antimicrobial exposure.

Our criteria for use align with a growing body of institutions aiming for targeted, risk-stratified use of fidaxomicin rather than unrestricted, first-line adoption. The United States Veterans Health Administration updated their fidaxomicin criteria for use to be nearly identical to our own [20]. Other institutions have similarly developed restriction criteria, but none have to our knowledge examined the impact on recurrence or any other clinical metric [21,22]. Our criteria were developed using comparable risk-based logic grounded in available evidence, identifying advanced age, immunocompromised status, prior recurrence, and ongoing or recent antimicrobial exposure as dominant drivers of recurrent CDI [5,6,7,8,9]. We intentionally required patients aged 65 or older to have an additional risk marker of concurrent systemic antimicrobial therapy rather than relying on age alone, as age in isolation is a comparatively weak discriminator [5,6,8]. When combined with concurrent antibiotic exposure, a well-established disruptor of the gut microbiota and a major contributor to recurrence, this approach may more precisely identify patients with compounded risk. Since the publication of the 2021 IDSA/SHEA-focused update, recurrence-prevention strategies have expanded beyond antibiotic selection (i.e., bezlotoxumab, fecal microbiota spores, live-brpk, etc.). While newer options may not be universally accessible because of cost and availability, their emergence reinforces the need for risk-based approaches to allocate high-cost interventions efficiently. This approach is also supported by parallel studies demonstrating that criteria-based restriction strategies for other high-cost medications, including intravenous immunoglobulin and colony-stimulating factors, have successfully reduced utilization without worsening clinical outcomes, suggesting that this stewardship model is generalizable beyond a single drug class [23].

Evidence from other studies informed our decision to embed fidaxomicin use criteria directly into the prescribing workflow with pharmacist verification, rather than requiring formal infectious disease consultation or relying on guideline dissemination and provider education alone. Khadem and colleagues demonstrated that, following a clinical guideline update at a large health system, only 50% of fidaxomicin regimens across ten community hospitals adhered to the revised institutional guidelines. Notably, infectious disease specialists were among those frequently non-adherent, underscoring that guideline dissemination alone is insufficient to change prescribing behavior even among specialists [22]. This observation is consistent with a broader stewardship study demonstrating that provider education and feedback, while valuable as components of comprehensive programs, produce inconsistent effects on antimicrobial prescribing metrics and may not lead to sustainable behavior changes when used in isolation [24,25,26]. In addition, implementation of fidaxomicin use criteria supports timely initiation of appropriate therapy when infectious disease specialists are unavailable, a situation that may otherwise result in treatment delays, by embedding pre-specified criteria into the order set with pharmacist verification.

The inclusion and exclusion criteria of this study were carefully designed to compare the recurrence rates of CDI pre- and post-implementation of the fidaxomicin use criteria between a comparable study population. Herein, this study excluded the patient population with rCDI since previous recurrence is consistently the strongest predictor of subsequent rCDI in the published literature [5,6,7,8]. Conversely, patients with severe CDI were excluded, as these patients were to receive oral vancomycin and intravenous metronidazole with or without vancomycin enema based on the local protocol. Exclusion of these patients allowed the matched analysis to more precisely isolate the effect of the criteria on the population in whom utilization was anticipated to change. However, this approach likely lowered the baseline recurrence risk compared with a CDI population with severe and recurrent disease included. Accordingly, our findings should be interpreted in this context, as the 90-day recurrence rate in the control group was only 7.3%, which is substantially lower than the 20–30% rates reported in the literature.

However, several limitations need to be considered for this study. First, the retrospective observational design introduces the potential for residual confounding despite the use of matching techniques in the electronic health record, including medication adherence, drug interactions, and other lifestyle factors that may influence recurrence independent of the index antimicrobial selected. Indeed, some of these factors may have been disproportionately present in the patients excluded after matching. The most profound risk factors were, however, included in the matching process or were not different between the unmatched and matched cohorts. Second, although the matched cohort achieved adequate power for the primary non-inferiority comparison, the post-implementation intervention group was relatively small (*n* = 48) and limited by project completion requirements. This incongruity may limit our ability to detect smaller but still clinically meaningful differences in recurrence. Third, both the criteria for use and the matching variables relied on risk factors identifiable from structured EHR data including age, immunocompromised status, and antibiotic exposure and could not incorporate other recognized but less consistently documented predictors of recurrence, such as CDI strain type, fecal microbiota composition, or detailed comorbidity staging, to name a few. Finally, the exclusion of patients without follow-up within our health system may limit the generalizability of our findings and introduce the potential for selection bias. However, this approach was necessary because recurrent CDI could not be reliably assessed in patients with no subsequent contact within our system. Patients with internal follow-up were included because recurrence events were frequently documented in clinical notes, even when diagnosis or management occurred outside our network.

Despite these limitations, our findings have important implications for other healthcare systems, particularly in a rural health area. The pharmacist-verified criteria for use built around a small number of well-established, EHR-extractable recurrence risk factors may help preserve access for patients considered at the highest recurrence risk while reducing overall utilization. Future prospective, multicenter studies with larger and more demographically diverse populations are warranted to validate these findings and further refine risk-based strategies for optimizing CDI management.

## 5. Conclusions

Implementation of fidaxomicin use criteria in a large rural health system was associated with reduced fidaxomicin utilization and lower antimicrobial expenditures without an observed increase in 28-day recurrent CDI in this matched cohort. These findings suggest that a risk-based stewardship approach may reduce costs while maintaining similar recurrence rates, although this study was not designed to definitively establish clinical equivalence. Our own criteria provide a practical framework for guiding stewardly CDI treatment decisions, but other healthcare institutions should adjust stewardship interventions to fit their own context and then confirm whether outcomes are maintained.

## Figures and Tables

**Table 1:** Fidaxomicin use criteria.

Must have both of the following (1 and 2):
Laboratory-confirmed symptomatic *C. difficile* infection; Patient has NOT already been started on oral vancomycin for this episode.
In addition to the required criteria, the patient must have at least one of the following 3 criteria (1, 2, or 3):
Patient is being treated for a recurrent episode of *C. difficile* infection; Patient is neutropenic or otherwise severely immunocompromised; Patient is ≥65 years old and is currently on systemic antibiotics.

**Table 2:** Summary of the overall and matched populations.

	All N = 174	Matched Sample N = 144
Age, mean ± sd	67.1 ± 14.3	66.5 ± 14.8
Sex, *n* (%)		
Female	87 (50.0)	72 (50.0)
Male	87 (50.0)	72 (50.0)
Facility, *n* (%)		
Flagship Hospital	84 (48.3)	68 (47.2)
Regional Hospital 1	34 (19.5)	26 (18.1)
Regional Hospital 2	20 (11.5)	17 (11.8)
Regional Hospital 3	12 (6.9)	12 (8.3)
Regional Hospital 4	11 (6.3)	9 (6.2)
Regional Hospital 5	6 (3.4)	6 (4.2)
Critical Access Hospital 1	3 (1.7)	2 (1.4)
Critical Access Hospital 2	2 (1.1)	2 (1.4)
Critical Access Hospital 3	2 (1.1)	2 (1.4)
CCI, median ± sd	3.0 ± 3.0	3.0 ± 2.9
Immunocompromised, *n* (%)	13 (7.5)	11 (7.6)
Antibiotics during index admission, *n* (%)	58 (33.3)	34 (23.6)
* Antibiotics 30 days prior to admission, *n* (%)	77 (44.3)	57 (39.6)

* Systemic antimicrobials as risk factors for development of CDI, not including oral vancomycin or fidaxomicin.

**Table 3:** Summary of the matched population.

	Control N = 96	Intervention N = 48	*p*-Value
Age, mean ± sd	66.3 ± 15.4	66.9 ± 13.6	0.801
Sex, *n* (%)			0.216
Female	44 (45.8)	28 (58.3)	
Male	52 (54.2)	20 (41.7)	
Facility, *n* (%)			0.322
Flagship Hospital	43 (44.8)	25 (52.1)	
Regional Hospital 1	17 (17.7)	9 (18.8)	
Regional Hospital 2	12 (12.5)	5 (10.4)	
Regional Hospital 3	8 (8.3)	4 (8.3)	
Regional Hospital 4	7 (7.3)	2 (4.2)	
Regional Hospital 5	6 (6.2)	0 (0.0)	
Critical Access Hospital 1	1 (1.0)	1 (2.1)	
Critical Access Hospital 2	0 (0.0)	2 (4.2)	
Critical Access Hospital 3	2 (2.1)	0 (0.0)	
CCI, median ± sd	4.0 ± 3.2	3.0 ± 1.9	0.051
Immunocompromised, *n* (%)	7 (7.3)	4 (8.3)	>0.99
Antibiotics during index admission, *n* (%)	23 (24.0)	11 (22.9)	>0.99
* Antibiotics in 30 days prior to admission, *n* (%)	36 (37.5)	21 (43.8)	0.588

* Systemic antimicrobials as risk factors for development of CDI, not including oral vancomycin or fidaxomicin.

**Table 4:** Antibiotic use and expenditure.

	Control N = 96	Intervention N = 48	*p*-Value
Antibiotic Use			<0.001
Vancomycin, *n* (%)	57 (59.4)	44 (91.7)	
Fidaxomicin, *n* (%)	39 (40.6)	4 (8.3)	
Antimicrobial expenditure	Control (2023 only)	Intervention (2025 only)	Relative Change (%)
Vancomycin (USD)	$6768	$7838	15.8%
Fidaxomicin (USD)	$165,964	$75,124	−54.7%

**Table 5:** Recurrence rate of CDI before and after implementation of the fidaxomicin use criteria.

Recurrence Rates	Control N = 96	Intervention N = 48	*p*-Value
28-day recurrence, *n* (%)	5 (5.2)	3 (6.2)	0.014
90-day recurrence, *n* (%)	7 (7.3)	5 (10.4)	0.079

## Data Availability

The data presented in this study are available upon request from the corresponding author due to the nature of the health information included in the raw data set.

## Supplementary Materials

The following supporting information can be downloaded at https://www.mdpi.com/article/10.3390/idr18040090/s1, Fidaxomicin Stewardship Strategy.

## Abbreviations

The following abbreviations are used in this manuscript:

CDI	*Clostridioides difficile* infection
rCDI	Recurrent *Clostridioides difficile* infection
CDNAAT	*Clostridioides difficile* nucleic acid amplification test
IDSA	Infectious Diseases Society of America
SHEA	Society for Healthcare Epidemiology of America
CCI	Charlson Comorbidity Index
EHR	Electronic Health Record
IV	Intravenous
