# Supplementary material for: The Impact of Hospital-Based Fidaxomicin Use Criteria on Clostridioides difficile Recurrence: A Retrospective Matched Cohort Study
Source: Infect Dis Rep. 2026 Aug 19;18(4):90. doi: 10.3390/idr18040090 (PMC13513588; doi:10.3390/idr18040090)
Supplement: Supplementary file 1 [file idr-18-00090-s001.zip › idr-4451950-supplementary.pdf]

ANTIMICROBIAL STEWARDSHIP PROGRAM

Fidaxomicin

Criteria for use pathway for treatment of laboratory-confirmed *Clostridioides difficile* infection (CDI).

Order set: **MED Adult C Diff Management** Review point: **Pharmacist, prospective**

Escalation: **ID consult**

1

ORDER ENTRY

Provider initiates the order set

Treatment is ordered through the standardized C diff management order set, which routes every fidaxomicin order through criteria-for-use review.

`MED Adult C Diff Management`

2

REQUIRED CRITERIA

All conditions below must be met

- [ ] Laboratory-confirmed, symptomatic CDI

AND

- [ ] No oral vancomycin started for the current episode

AND

- [ ] AT LEAST ONE OF THE FOLLOWING
  - Recurrent CDI
  - Severely immunocompromised or neutropenic
  - Age ≥ 65 years **and** receiving systemic antibiotics

3

PHARMACIST REVIEW

Fidaxomicin order submitted

Pharmacist performs prospective review against the same criteria-for-use before the order is verified.

DECISION

CRITERIA MET

Verify without intervention

Order proceeds as written; no provider contact required.

CRITERIA NOT MET

Contact provider

Pharmacist reaches out and recommends oral vancomycin in place of fidaxomicin.

ACCEPTS

Switched to oral vancomycin. No further action.

INSISTS

ID consult required before the order can be verified.
